# Modelling cortical network dynamics

**DOI:** 10.1007/s42452-024-05624-8

**Published:** 2024-01-29

**Authors:** Gerald Kaushallye Cooray, Richard Ewald Rosch, Karl John Friston

**Affiliations:** 1 Clinical Neuroscience, Karolinska Institutet, Stockholm, Sweden; 2 GOS-UCL Institute of Child Health, University College London, London, UK; 3 The Wellcome Centre for Human Neuroimaging, Queen Square Institute of Neurology, University College London, London, UK; 4 MRC Centre for Neurodevelopmental Disorders, Institute of Psychiatry, Psychology and Neuroscience, King’s College London, London, UK

## Abstract

We have investigated the theoretical constraints of the interactions between coupled cortical columns. Each cortical column consists of a set of neural populations where each population is modelled as a neural mass. The existence of semi-stable states within a cortical column is dependent on the type of interaction between the neuronal populations, i.e., the form of the synaptic kernels. Current-to-current coupling has been shown, in contrast to potential-to-current coupling, to create semi-stable states within a cortical column. The interaction between semi-stable states of the cortical columns is studied where we derive the dynamics for the collected activity. For small excitations the dynamics follow the Kuramoto model; however, in contrast to previous work we derive coupled equations between phase and amplitude dynamics with the possibility of defining connectivity as a stationary and dynamic variable. The turbulent flow of phase dynamics which occurs in networks of Kuramoto oscillators would indicate turbulent changes in dynamic connectivity for coupled cortical columns which is something that has been recorded in epileptic seizures. We used the results we derived to estimate a *seizure propagation* model which allowed for inversions using the Laplace assumption (Dynamic Causal Modelling). The seizure propagation model was trialed on simulated data, and future work will investigate the estimation of the connectivity matrix from empirical data. This model can be used to predict changes in seizure evolution after virtual changes in the connectivity network, something that could be of clinical use when applied to epilepsy surgical cases.

## Article Highlights

- We investigate in-silico models of cortical networks generating complex activity seen in data from animals and humans.
- We propose an in-silico model that links network structure to epileptic seizures propagation.
- This work presents a technical pipeline that can be used in future studies of epileptic networks.

## Introduction

The human cortex consists of a lattice of cortical columns with dense intracolumnar connection and a sparse network of intercolumnar connections [1]. The histological structure of the cortex consists of several layers of different types of neuronal cells with different structures and receptors [2]. Neuronal activity within each column generates extracellular currents whose effects can be measured using electrodes and sensors in the near vicinity and more coarsely at relatively great distances outside the human body using scalp electro- and magnetoencephalography (EEG and MEG) [3]. These recordings consist of broadband frequency activity; the frequency content is partly shaped by the different types of postsynaptic receptors present. The cortical activity also indicates stochastic features of the underlying dynamics, including spikes, sharp transients, and paroxysmal rhythms seen both in the healthy and dysfunctional brain [3].

Key features of the dynamics of cortical column activity have been described by a multitude of generative models, ranging from one-dimensional integrate and fire neurons, multidimensional neuronal mass models and infinite dimensional partial differential equations. Neuronal mass models allow for sufficient simplification of the cortical columns to allow for analytically tractable dynamics of cortical networks [4–6]. The simplest neural mass models are the convolution-based models where synaptic kernels are used to convolve presynaptic input to produce postsynaptic dynamics. The canonical microcircuit model typifies a convolution based neural mass model of a cortical column and consists of 4 interacting excitatory/inhibitory neuronal populations with recurrent coupling [7]. The dynamics of the cortical column are given by 4 coupled 2nd order differential equations. The possible trajectories of such a system can be characterized using the phase space representation [8]. Through simulation, these systems have been shown to have complex structures in phase space including stationary points, limit cycles and chaotic attractors [9–11]. Perturbative activity at a stable point is often used to model spontaneous activity as seen on EEG and MEG recordings [12–14]. However, when system behavior changes dynamically over time, i.e., iterant activity, it would be better modelled using the full set of dynamics involving different semi-stable sets such as limit cycles and stationary points [10]. The biological interpretation of limit cycles would be high amplitude oscillatory activity including physiological and abnormal activity including epileptic activity like seizures and spikes. We have previously published (under specified conditions) the sufficient and necessary constraints required for a neural mass system to have limit cycles [15]. In these models, the type of coupling between neuronal subpopulations determines the stability of the topology of the phase space structure i.e., the presence of different stable dynamic regimes. In our previous study we showed that synaptic kernels of potential-to-current coupling (S-coupling) will only affect the phase of the trajectories of the model keeping the topology constant, i.e., without any effect on the stability of limit cycles. In contrast, synaptic kernels of current-to-current coupling (P-coupling) will affect the stability of limit cycles, as does cross coupling between potential-to-current and current-to-current coupling (i.e. the interaction between S and P-coupling) [15]. The latter involves at least two neuronal subpopulations and allows for complex cross coupling between activity of different frequencies. Generative models with complex phase space topology, would be well suited for analysis of empirical data, with similar complexity including, time-varying, and paroxysmal brain dynamics. This would be specifically important for model-inversion schemes e.g., dynamic causal modelling (DCM).

We will continue using the perturbative analysis presented previously to investigate the interaction between cortical columns and deriving the equations of motion for this activity [15]. Interestingly, our theoretical considerations of interacting neural mass models, allows us to derive the equations of motion for the phase of the cortical activity which turns out to be the much-celebrated Kuramoto model [16, 17]. The Kuramoto model has been used as a phenomenological model of cortical activity, but has not, to our knowledge been derived directly from more biophysical plausible models (e.g., neural mass models) [18–20]. Moreover, our derivations reveal a complex interaction between columns where the transition between the semi-stable states of each column is determined by the connectivity between the columns as well as the phase difference in activity. Thus, intercortical connectivity is best described through two terms, a static connectivity and a dynamic phase-dependent connectivity. Epileptic seizures have been identified as brain states with dynamically changing connectivity although a clear understanding of the underlying dynamics is still not well understood. Different studies have presented conflicting results on connectivity changes during the onset, propagation and termination of a seizure [21–23]. We used our findings to construct a model of seizure propagation, allowing for inversion techniques (DCM) to estimate the connectivity matrices from EEG recorded during epileptic seizure onset and propagation. The theoretical work presented in this paper deepens our understanding of dynamical itinerancy and will also provide tractable ways of incorporating our knowledge in schemes of model inversion (e.g., DCM).

In Sect. 2 we will summarise and further investigate the model used to analyse cortical columns. In Sect. 3 the interactions between cortical columns are analysed together with simulations and a proposed seizure propagation model. In Sect. 4 we will discuss the findings of the study followed by Sect. 5 giving the mathematical background for Sect. 2 and 3. References are given in Sect. 7.

## Summary of previous results

### Cortical column dynamics

We will present a summary of the results derived in [15]. Please see the Appendix (5.1–3) for a full mathematical derivation of the results in this section. The equation of motion of the neural mass model is given by,

2.1 $$\dot{{p}_{i}}=-{{\omega }_{i}}^{2}{q}_{i}+\varepsilon {{\omega }_{i}}^{2}\sum {s}_{ij}S\left({q}_{j}\right)+\mu {{\omega }_{i}}^{2}\sum {p}_{ij}P\left(\frac{{p}_{j}}{{\omega }_{j}}\right)$$

$$\dot{{q}_{i}}={p}_{i}$$

The S-coupling represent the synaptic potential to current coupling and the P-coupling the synaptic current to current coupling, both of these coupling types are commonly present in neural mass models [7]. Note that the S-coupling is positive close to 0 and the P-coupling negative. These equations will give the standard dynamics of a neural mass model and it can be re-written using complexification which will simplify the subsequent derivations. Note that connections between neuronal units are dependent on axonal (or dendritic) pathways between the units and the types of synaptic interactions involved. As synaptic interactions can both increase or decrease a membrane potential, we have allowed the connection parameters to take both positive and negative values. The complexified equation of motion will be given by the following,

$${\dot{z}}_{i}=-i{\omega }_{i}{z}_{i}+i\varepsilon {\omega }_{i}\sum {s}_{ij}S\left(\frac{{z}_{j}+{{z}_{j}}^{*}}{2}\right)+i\mu {\omega }_{i}\sum {p}_{ij}P\left(\frac{{z}_{j}-{{z}_{j}}^{*}}{2i}\right)$$

The complex variable, z, is given by the following combination of the current and potential variable,

$${z}_{i}={q}_{i}+i\frac{{p}_{i}}{{\omega }_{i}}$$

**Fig. 1:**
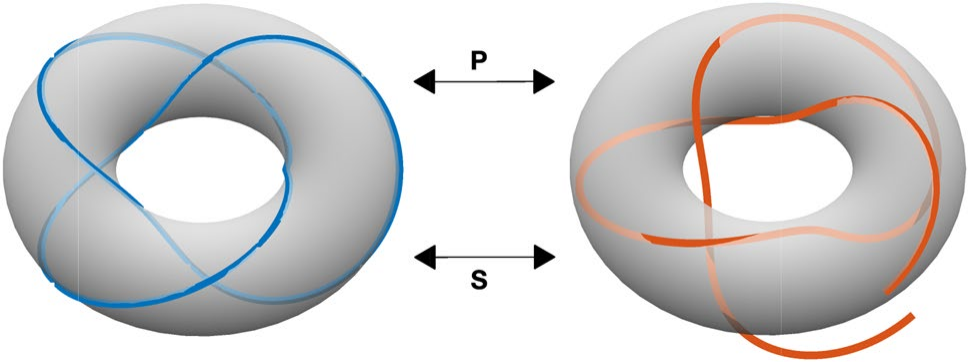
Schematic image of the trajectory of two interconnected neuronal populations. The fast dynamics is given by the frequencies of the individual neuronal populations (in the figure it is of the order of 0.3 time-units). The slow dynamics is determined by the time it takes for a trajectory to complete one full cycle looping back (or almost back) to the starting point (which is of the order 1 time-units). With zero coupling the trajectories loop back on themselves as shown to the left (blue) and with a perturbation there is a lag as shown to the right (red). This lag can be estimated using a perturbation series expansion

The dynamics with zero coupling between N neuronal populations (ε = μ = 0) will give trajectories on a N-dimensional torus, for two neuronal populations the activity will be on a doughnut-shaped surface, Fig. 1. There are several time scales of the dynamical activity, the fastest of these scales is the period of oscillation for each neuronal population which is determined by their intrinsic frequencies. However, as can be seen in the figure the trajectories with zero coupling (in blue) loop back on themselves and the period for this is often on a larger time scale than the individual periods of the neuronal populations. When a perturbation is added to the dynamics the trajectories will leave the toroidal surface and not loop back as shown in the figure (in red). This deviation can be estimated using a perturbative series expansion and will have both an amplitude and phase term.

The dynamics can be simplified by averaging over the fast variables leaving the slow dynamics using an adiabatic approximation [8, 24]. To perform the adiabatic approximation the activity of the neural masses is integrated over the periods (or near periods of the original activity). The instantaneous amplitude R will be replaced by an averaged amplitude over cycles and the instantaneous phase of the dynamics will be replaced by the resulting phase lag when completing a (near) period, Fig. 1. For small couplings a perturbative series expansion can be done to analyse the effects of interpopulation coupling. The changes in the amplitude variable of the trajectory after integrating over a full cycle (defined subsequently as the derivative of the flow in the new time scale) is only affected by the *P*-coupling (when including terms up to 1st order in the perturbations series), see Sect. 5.2-3 and Eq. (5.2.2). The S-coupling will result in a change of phase lag per cycle and including both types of coupling will give an additional cross coupling (S coupling followed by P coupling or vice versa) but of much lower magnitude [15].

2.2 $$\frac{d{R}_{i}}{dt}={\omega }_{i}{p}_{ii}f({R}_{i})$$

**Fig. 2:**
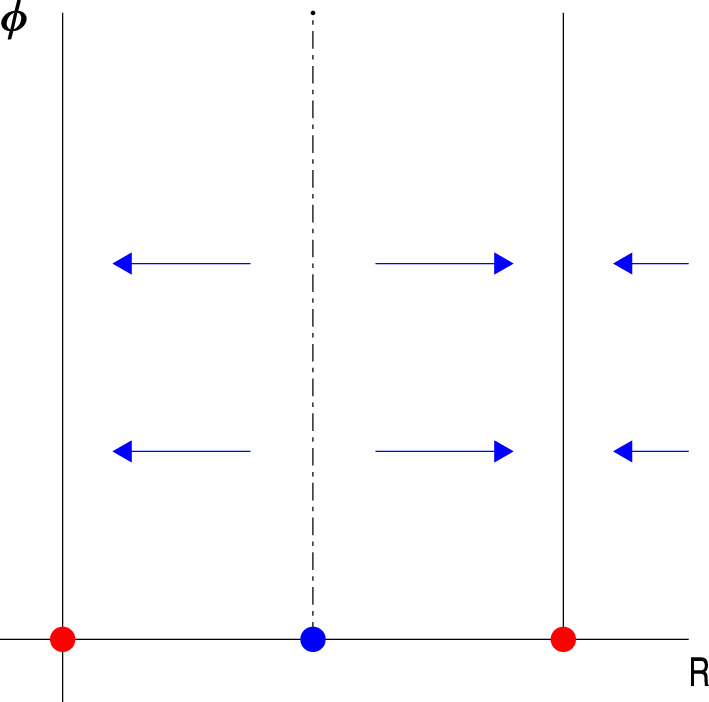
Adiabatically approximated dynamics show at least one stable point (red) at the origin. Depending on the shape of the synaptic kernel of type P there could be more stable points. The simplest non-trivial shape will have 1 stable (red) and unstable limit cycle (blue) as shown in the figure. Note that there is no dependence on the phase (ϕ) but only on the amplitude (R)

The phase portrait is dependent on changes in amplitude but not directly on changes in phase, and the stable points of Eq. (2.2) (i.e., when the right-hand side is 0) will define the limit cycles of the original dynamics, see Fig. 2. The stable points of Eq. (2.2) will be determined by the shape of the synaptic P-kernel. A monotonic P-kernel will only have a stable point at 0, and not give rise to any limit cycles. The simplest non-trivial shape for the P-kernel will have a stable point at 0 and 2 limit cycles (one stable and one unstable).

### Interaction between off diagonal and diagonal terms within a cortical column

**Fig. 3:**
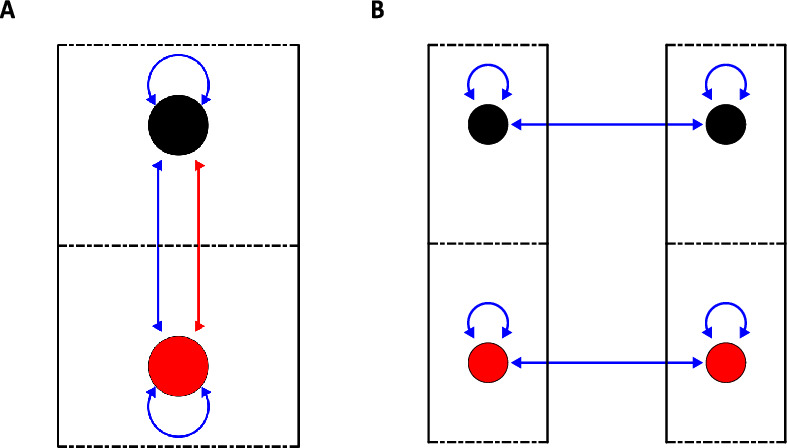
A. A schematic figure of one cortical column with two neuronal populations (black ball in the upper layer and red ball in the lower layer). The self-interactions are given by P-coupling (blue arrows) and the connections between the two layers is given by S- (red arrows) and P-coupling. The S and P connections strengths when varied will result in different dynamics. B. A schematic figure of two cortical columns with two layers each (with same coloring as in 3A). The dynamics of the full system will be determined by the self-interaction and extrinsic connections between homologous neuronal populations. Interactions between different types of neural populations will consist of second order terms or higher in the perturbation series and are therefore not included. The cortical layers are effectively decoupled

In this section we will investigate and contrast the effect of self-coupling (represented by diagonal terms of the connectivity matrix) and coupling between different subpopulations within a cortical column (represented by off diagonal terms of the connectivity matrix). The analysis is done on one cortical column with two layers containing both P and S coupling, Fig. 3).

**Fig. 4:**
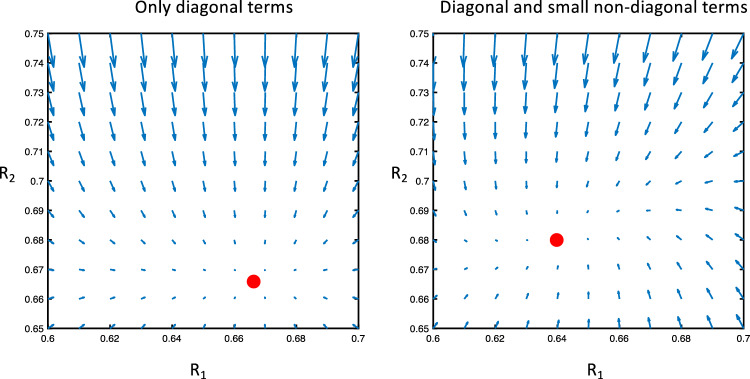
Interaction between 2 neuronal subpopulations is estimated where the amplitude variables for 2 populations in one cortical column are shown (R_1_ and R_2_). The vector plot around the stable point for different off-diagonal couplings is shown with no off-diagonal terms to the left and small off-diagonal terms to the right. Small off diagonal terms result in a small deviation of the flow but retains the stability of the stationary points which represents limit cycles in the original model. The red ball indicates a stable limit cycle (note that the phase variables are not included in the figure.)

The stable sets (stable points and limit cycles) of an uncoupled system of neuronal populations will retain stability when coupled using small interactions terms as shown in Fig. 4 (see Sect. 5.2 for details on the variables used to estimate the dynamics).

**Fig. 5:**
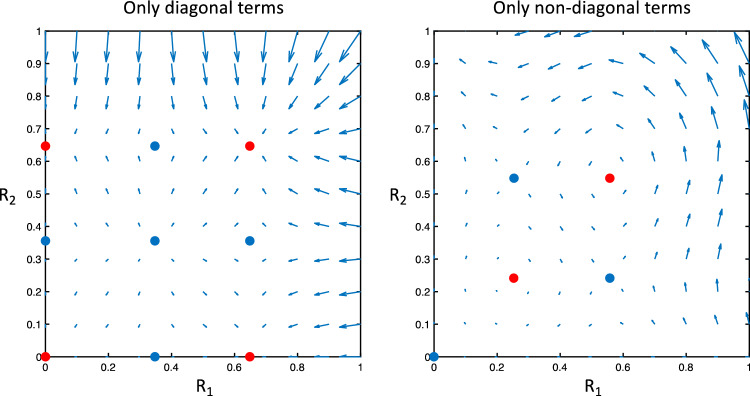
Vector plot describing phase space dynamics for different connectivity matrices. The amplitude variables for 2 populations in one cortical column are shown (R_1_ and R_2_). The panel to the left shows 4 stable (in red) and 5 unstable stationary points (in blue) where the off-diagonal components have been set to 0. The stationary points would represent stable or unstable limit cycles of the original model, except the stationary point at zero which would translate to a stationary point of the original model (i.e., the model before the adiabatic approximation). The panel to the right shows the dynamics with only off-diagonal terms in the connectivity matrix. 2 stable (in red) and 3 unstable stationary points (in blue) are seen representing limit cycles (or stationary point at the 0) of the original model

The dynamics (phase-portrait) of a 2-layer cortical column is quite different for large interaction terms in comparison to small with a change in the number of limit cycles and their stability as shown in Fig. 5 (see Sect. 5.2 for details on the variables used to estimate the dynamics).

The trajectories for off-diagonal coupling within a cortical column increases in complexity as the number of limit cycles per cortical column increases as the trajectories start folding tightly around each other. The equation for the dynamics is derived in Sect. 5.2, Eq. (5.3.2).

## Interaction between columns

In this section we will investigate the interaction between cortical columns estimating the change in activity of each column due to mutual coupling. We will further show that the interaction between cortical columns is not only determined by the coupling between them but also their phase difference (see 3.1 for details) where the interaction will be given by the Kuramoto model (see 3.2 for details). Using the results from 3.1 to 2 we will derive the stability of semi-stable states, i.e., the transition times from semi-stable states to global states of stability (see 3.3 for details). We further, estimate robust methods for model inversion, estimating the connectivity matrix from transition times (3.4). Finally, all the results from 3.1–4 are combined and used to derive a model for estimation of intercortical connectivity from seizure propagation in Sect. 3.5. Inversion of the synthetic data was performed using standard (variational Laplace) inversion routines available in the SPM software package. The code used in this paper can be obtained from https://github.com/gercoo/sz_propagation.

### Interaction between 2 cortical columns

We will investigate the effect of S and P coupling between two interconnected cortical columns and get a new set of dynamics for the columns after the adiabatic approximation. Interlayer connections give second order or higher terms in the perturbative expansion which allows us to effectively decouple the cortical layers [14]. The interaction was determined (including 1st order terms in the perturbative series) by self-interaction terms (g_ii_) for a single column and connections between the same layers of the two cortical columns (h_ii_), Fig. 3, (Sect. 5.3).

As the cortical layers are effectively decoupled, we will perform the rest of the analysis on a single cortical layer. The semi-stable states of a single layer of a cortical column are given by the stationary points of Eq. (2.2) which in the simplest case of multiple semi-stable states would be a stationary point at 0 and a stable limit cycle (of amplitude *R*_*s*_) which will be indexed as follows,

$$\left\{0\right\},\{{R}_{s}\}$$

Each layer of two interconnected columns will have the following stable states for weak extrinsic connection (where the first entry indicates the stationary state of the first column and the second entry that of the second column),

$$\left\{\mathrm{0,0}\right\}, \left\{0,{R}_{s}\right\},\left\{{R}_{s},0\right\},\left\{{R}_{s},{R}_{s}\right\}$$

**Fig. 6:**
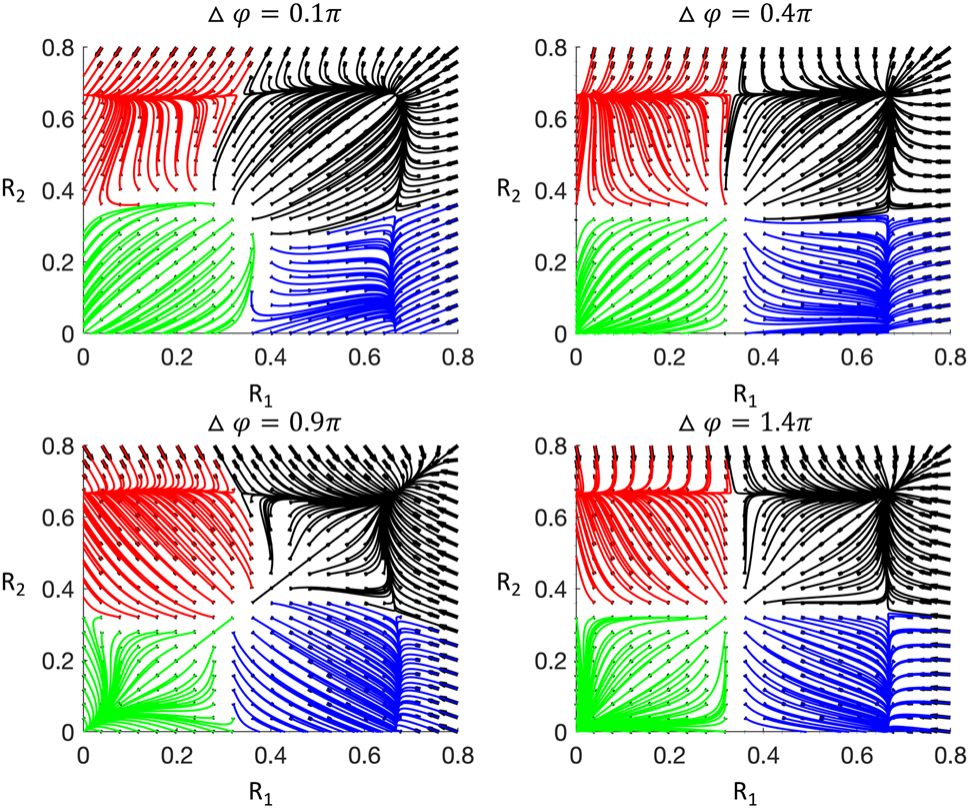
Phase space plots with trajectories for different values of phase lag (Δφ). The amplitude variables for a given layer of the two connected cortical columns are shown (R_1_ and R_2_). There are 4 stable states and the trajectories which are attracted to each stable point are drawn with a different colour. Each stable point will represent a limit cycle in the dynamics of the cortical column (or stable point at 0). The geometry of the phase portrait is dependent on the phase lag (as can be seen in panel A, B, C and D), although the topological structure does not change

As detailed in Sect.5.3–4 the phase lag between the two columns will determine the trajectories together with the amplitudes of the activities of the neuronal populations. The phase portraits have the same topological structure but the geometry of the phase portrait is is dependent on the phase lag, Fig. 6.

We will investigate the following state transition:

$$\left\{0,{R}_{s}\right\}\to \left\{{R}_{s},{R}_{s}\right\}$$

**Fig. 7:**
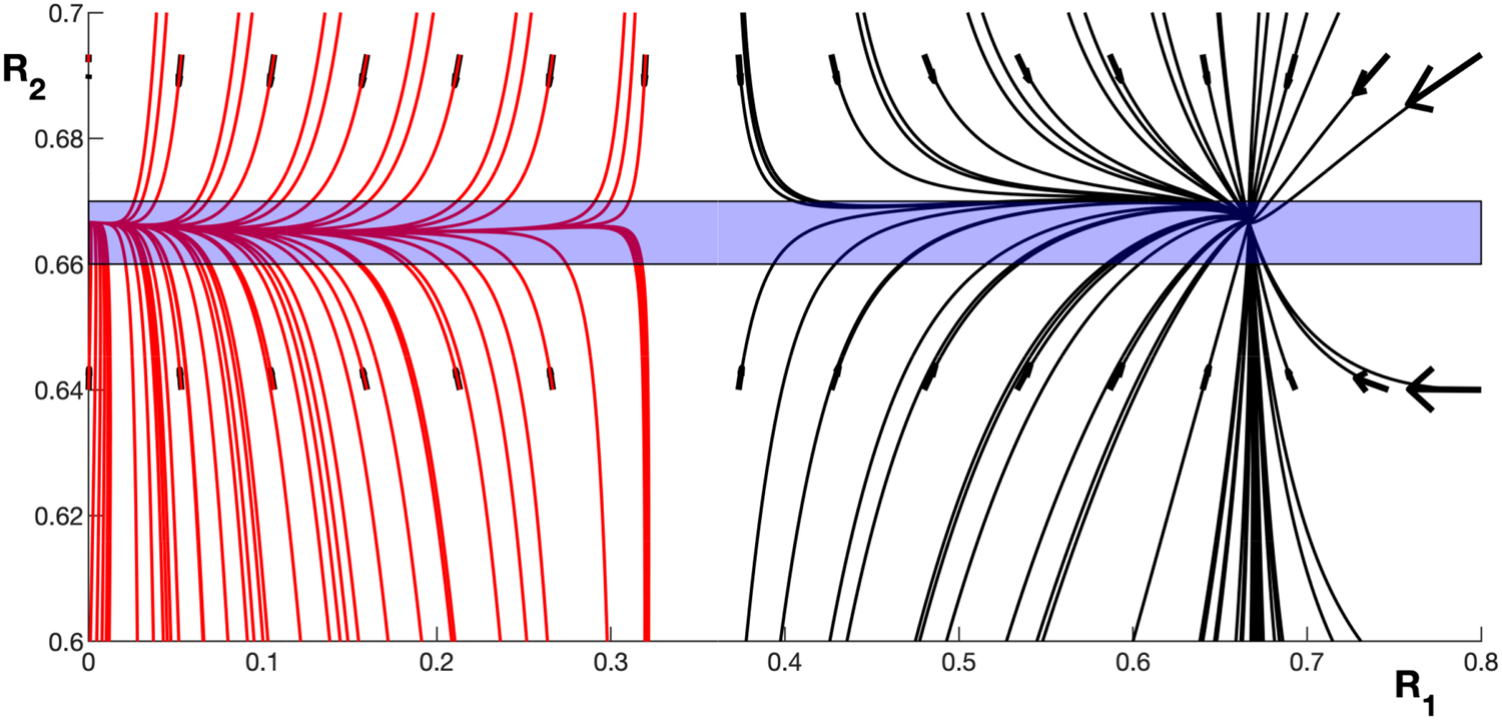
Phase portrait of trajectories flowing to the stable states {0, R_s_} (red lines) and {R_s_, R_s_} (black lines). The amplitude variables for a given layer of the two connected cortical columns are shown (R_1_ and R_2_). The transition pathway between the stable states can be approximated to occur within the blue shaded region as the flow of the dynamics is approximately pointed towards the line R_2_ = R_s_

The trajectory between the two states will be dependent on both the amplitude and phase lag variables and these variables will interact dynamically. However, to keep the results tractable we will decouple the phase lag from the amplitude variables. Adding a noise process to the system would randomly push trajectories from the paths plotted in the phase portraits. However, the actual trajectories (with ongoing noise) between the two stable states will occur close to the constant line R_2_ = R_s_ as can be seen in Fig. 7. Deviations of a trajectory from this line will rapidly return as the flow is pointed towards the line.

**Fig. 8:**
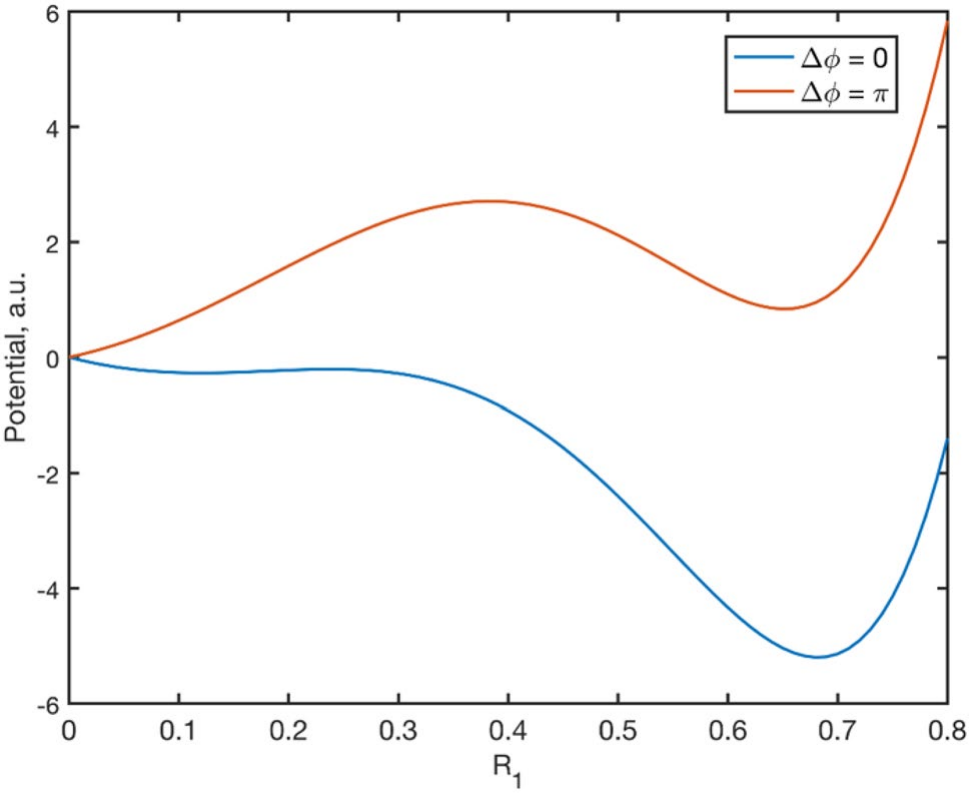
The trapping potentials for different values of phase lag are depicted in the figure. When the phase lag between the two cortical columns is 0, the stable point at {R_s_, R_s_} has global stability; when the phase lag is π, the global stability is at {0, R_s_}

Integrating the flow along R_1_ will give us the potential trapping the trajectories near the stable points. The potential is dependent on the phase lag between the cortical columns and noise process affecting the columns, Fig. 8. Phase differences close to 0 will have a global stable point at {R_s_, R_s_} while phase differences close to π will have a global stable point at {0, R_s_}.

The transition rates between semi-stable states for a given network of cortical columns will depend on the phase lag between the columns (and the coupling) allowing for the definition of a dynamic connectivity between cortical columns. This could play a considerable role in seizure propagation and the dynamic nature of connectivity indicated in experimental data where various connectivity measures between different cortical regions vary in time, especially during seizure progression. We propose with the current analysis that the dynamic variation in connectivity seen in empirical data is at least partly dependent on the phase of ongoing neuronal oscillations.

### Phase dynamics

The phase and amplitude dynamics are coupled; however, as an approximation (mainly to keep the results tractable as was mentioned in 3.1) they can be uncoupled using the average amplitude values (averaged over different trajectories). The phase dynamics will then be given by the following equation where C is a constant (Sect. 5.4),

3.2.1 $$\frac{d}{dt}{\varphi }_{i}^{1}=C{\text{sin}}({\varphi }_{i}^{1}-{\varphi }_{i}^{2})$$

This equation will approximate the activity around the stationary sets (stationary point or limit cycles) of the cortical columns. It is equivalent to the celebrated Kuramoto model and can also be derived from the Hamiltonian of the XY-model [25]. This model has been used to describe phase transitions in solid-state physics and has been shown to have interesting topological phase transitions as was shown by Berezinskii, Kosterlitz and Thouless [26]. Systems close to local equilibrium states as approximated in equation Eq. (3.2.1) will also have excitations with energies approaching 0, indicating long range correlations at low temperatures (goldstone modes or bosons) which were first described in the analysis of superconductivity by Nambu and Goldstone [27, 28]. The presence of low energy excitations allows for spatial correlations to take place over large spatial distribution in the cortex which could be of importance in the collation of data in the human brain.

### Simulations of state transitions

**Fig. 9:**
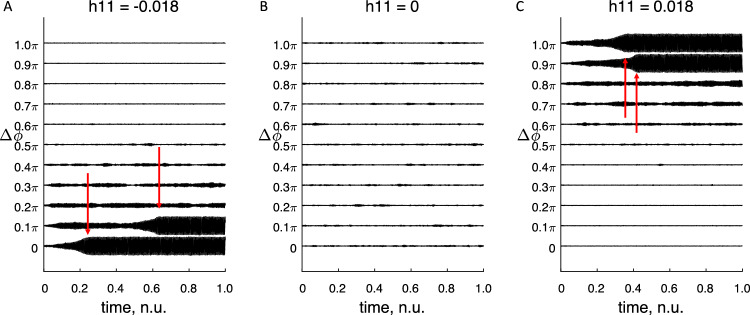
**A** Activity of a cortical column (with a semi-stable state at the origin and a limit cycle) when under the influence of a second cortical column oscillating in the limit cycle state. The activity will for small phase lags transit to limit cycle oscillations (with an increase in amplitude of the oscillation as seen on the plotted graphs). The time for transition from small amplitude activity to large amplitude activity is seen to depend on the phase lag between the columns. **B** With no connection between the columns (h_11_ = 0) there will be no transition to the limit cycle oscillation during the time plotted. **C** Similar activity as A but the connection between the cortical columns is “switched on” for phase lags corresponding to π and is “switched off” for phase lags around 0. Note the opposite sign of intercolumnar connection for A and C (h_11_). The time for full state transition is marked with red arrows. An interval of connectivity values around h_11_ = 0 were tested which, showed similar results

The above derivations indicate that state transitions are dependent on the phase difference between columns. To further investigate this statement, 2 cortical columns were interconnected and simulated (using the Euler-method and neural mass equations). The effects of intercolumnar connectivity and phase difference were studied. One column was allowed to oscillate at a limit cycle and did not receive feedback from the second column. The phase lag between the two cortical columns was fixed (not allowed to vary by adjusting the phase lag at each step of the simulation) to estimate the effect of connectivity coupling and phase lag on the dynamics of the second cortical column. With fixed connectivity between the columns the transition rate between the two semi-stable states of the second column was determined for different phase lags. The results indicate the existence of a dynamic and static connectivity where the transition time from one state to another is determined by the dynamic connectivity. This can relatively quickly be switched on or off depending on the phase lag between the interacting columns, Fig. 9. Note that the transition for negative connection strength occurs with small phase lags which is what could be expected during a seizure. The connection strength is negative and not positive (as could be expected) as the P-coupling has been defined such that it is negative close to 0.

Simulations and theoretical work indicate that larger networks of interacting systems produce a turbulent flow (in 2 dimensions) of phases where discontinuities are created by vortices [20, 25, 26]. The presence of similar vortices on cortical networks could cause rapid, perhaps turbulent, changes in phase lags which in turn would cause changes in the “dynamic” connectivity. This could alter the propagation pathways of cortical activity in an almost random fashion.

### Inverse modelling to determine synaptic intercolumnar connectivity

We will construct a forward model which will map the connectivity matrix to propagation times for the transition from low amplitude activity to high amplitude oscillations (proxy for seizure activity) for different cortical columns. The output will be the estimated time for the dynamics of a cortical column to transit between the semi-stable states. The potential driving the transition between the 2 stable states is estimated in Sect. 5.5. Adding noise to the system will cause random fluctuations in the trajectories and an initial probability distribution of starting points for trajectories will evolve according to the Fokker–Planck equation. The flow of the distributions could then be parameterised using the mean (μ) and the standard deviation (σ) of the true distribution. We will further assume that ${h}_{ii}>{g}_{ii}$ which will give the following relation,

$$\mu \left(t\right)=\mu \left(0\right)-{h}_{\mathit{ii}}{\text{cos}}\left({\varphi }_{i}^{1}-{\varphi }_{i}^{2}\right)t$$

This indicates that the transition rate between the low amplitude and high amplitude state is directly proportional to the value of *h* (the extrinsic connectivity) and dependent on the phase lag between the two columns (as was shown in the simulation in 3.2) and will only occur if the drift of the mean is positive (i.e., a drift from low amplitude to large amplitude activity). Note that when the cosine of the phase lag is near zero, the true transition time will be given by terms related to g_ii_, see Sect. 5.5 for the full expression for the transition rate. The transition time is defined as the time for the mean of the distribution to drift from the stable state at 0 to the attractor of the limit cycle.

### Modelling seizure propagation

Each cortical column will have two stationary states of activity, a stable point around the origin and a stable limit cycle. We will explore the spread of high amplitude activity (as a proxy for electrographic seizure activity) through a network of cortical columns. We will be investigating seizure onset and the connections of the network will be assumed to be cascading i.e., areas involved later in the seizure do not connect to areas involved earlier. Breaking this assumption would allow for the study of seizure termination, however, the complexity of the model increases and we will not pursue that further in this paper.

As a typical example we will simulate seizure onset times (*T*_*j*_) for N electrodes with an unknown connectivity matrix which will be estimated using the onset times and phase lag values between each electrode pair. The mean of the probability distribution of the trajectories can be estimated using the following formula (Sect. 5.5 and Eq. 5.5.2),

$$\mu \left(t\right)=\mu \left(0\right)-{h}_{\mathit{ii}}{\text{cos}}\left({\varphi }_{i,\mathrm{0,0}}^{1}-{\varphi }_{i,\mathrm{0,0}}^{2}\right)t$$

**Fig. 10:**
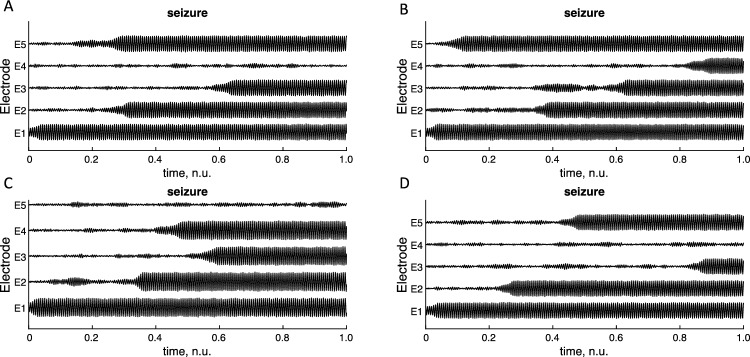
Seizure propagation simulated using 5 interconnected nodes. Note that seizure propagation varies for each trial even though the connectivity matrix is kept constant, illustrating the stochasticity of the propagation

The dynamics is determined by the connectivity matrix, ***h***, and phase lag between the cortical columns. To investigate the validity of these derivations, seizure spread was simulated using the neural mass model for interacting cortical columns as presented previously. The Euler method and an underlying noise process was used for the simulation. Seizure progression is not unique for a given connectivity matrix as this is determined by a mixture of deterministic and stochastic variables, Fig. 10.

**Fig. 11:**
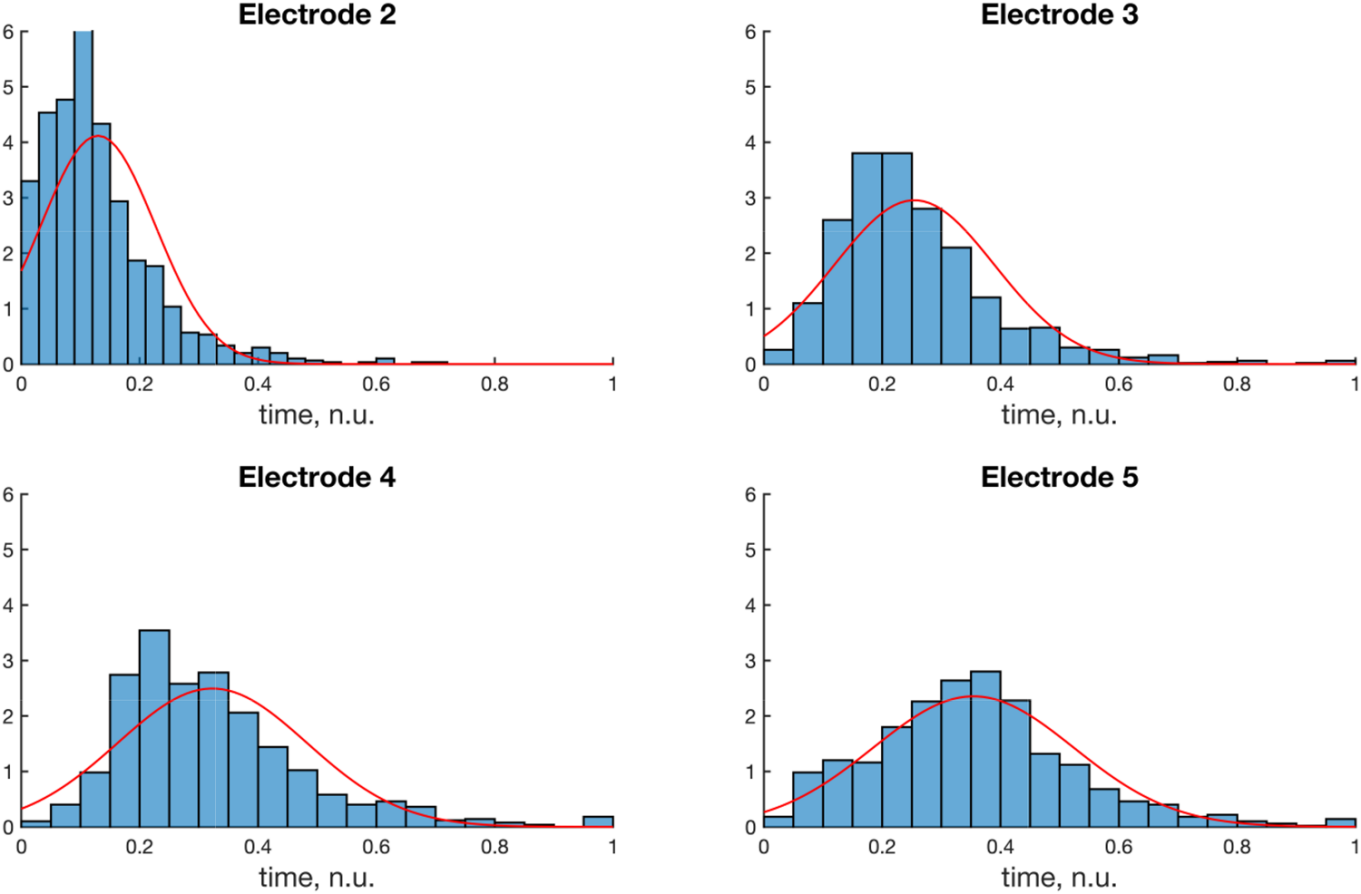
Probability distributions for seizure onset for five different electrodes (or cortical columns) with fitted probability distributions for the mean. Seizure propagation was simulated using the neural mass model for interacting cortical columns as presented previously. Five nodes were used in the network with seizure onset on electrode 1. The Euler method and an underlying noise process was used for the simulation. 1000 simulations were used and the onset times (in bins) were approximated using a normal distribution

The distribution of seizure onset for the same connectivity matrix was estimated using 1000 simulations of the same model with fitted normative distributions, Fig. 11.

**Fig. 12:**
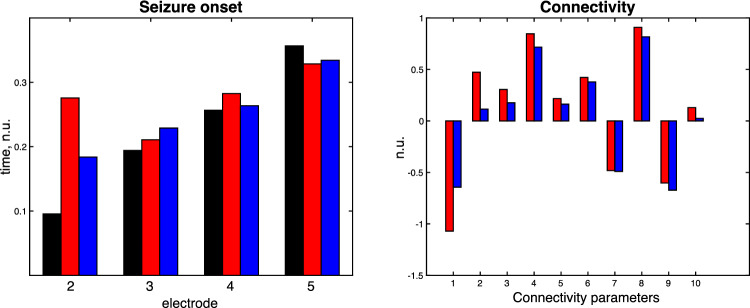
The results for seizure network inversion using Dynamic Causal modelling. A 5 network model was used to simulate seizure onset times (black) as described in Fig. 12. The seizure onset times were also estimated for a prior network (before inversion, red) and a posterior network (blue) after inversion. A comparison between the prior (red) and posterior (blue) parameters are given to the right, where the baseline values were normalised to 0. The posterior estimates of the connectivity values tend towards the baseline values (blue columns are in general smaller in amplitude than the red columns)

**Fig. 13:**
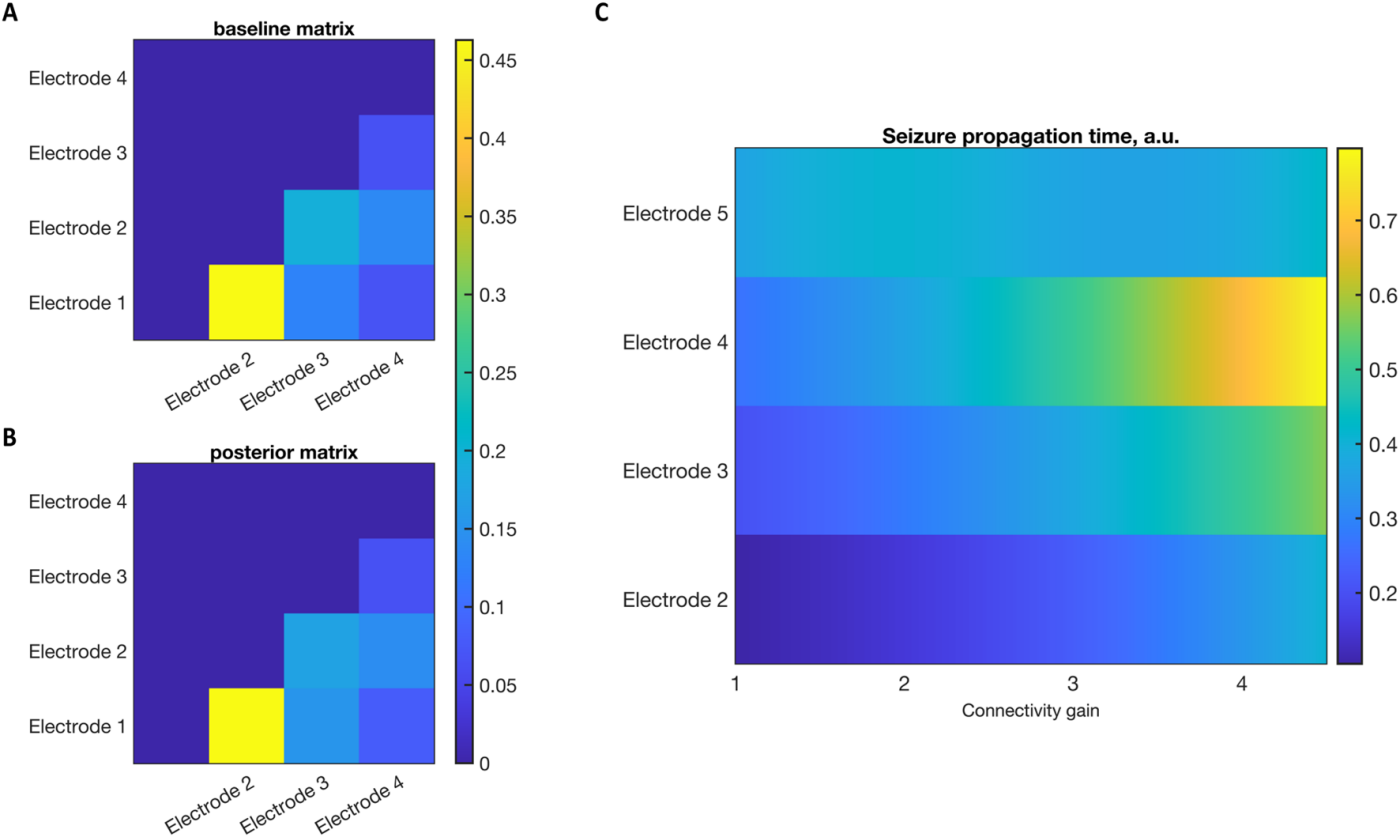
**A** Colormap of the baseline connectivity matrix with connection strengths between the network electrodes. **B** Colormap of the posterior connectivity matrix **C** Variation in seizure propagation time modelled by varying the gain of the connectivity matrix

Using only the summary statistics (mean and standard deviation) of seizure onset times across multiple observations, we inverted our model for seizure propagation (using DCM) (Sect. 5.5) estimating the connectivity matrix, see Figs. 12 and 13 for details of the inversion. In general, the estimated connectivity matrix (posterior estimates) was nearer the baseline values than the prior values. However, the change between the prior and the posterior distribution is determined by the variances of the prior estimates and also the risk of estimating posteriors at a local minima, factors that can be nuanced to the specific hypothesis being tested [29]. The evidence for the model is given by the free energy (-6.904) which could be used to compare different models of the same data. To investigate the effect of connection strength on the seizure propagation time we varied the latter and estimated the propagation time. As the connection strength was reduced there was an increase in propagation time (as shown in Fig. 13C). These changes could be of clinical interest, as a proxy for disconnecting the epileptogenic network, where the perturbation of the network led to a slowing of the seizure spread.

## Discussion and conclusion

In this study we continued our theoretical investigating into the dynamics of cortical columns, in particular the interaction between different columns. We have previously investigated the sufficient and necessary criteria for semi-stable states within a cortical column. It was shown to be dependent on the synaptic kernels connecting the neuronal subpopulations. The topological structure of the phase portrait was determined by synaptic kernels modelling current-to-current coupling. Moreover, it was shown that only those connections between neural subpopulations which led to a “resonance” of activity survived the adiabatic averaging and could affect the dynamics. For a single cortical column, resonating connections were given by self-connecting loops involving one or more neural subpopulations. These semi-stable states within a cortical column remained stable for small coupling between cortical columns. Even though intracortical (intrinsic) connectivity required self-connecting loops to give resonating terms, a mixture of intercortical (extrinsic) and intrinsic connectivity resulted in resonating terms from open ended connections (i.e., a connection between different neuronal subpopulations). This would happen if neural subpopulations from different cortical columns oscillating at the same frequency were connected (directly or through other types of neuronal subpopulations). The presence of open-ended connections introduced a new variable into the dynamics of cortical columns; the phase lag between identical neural subpopulations in different columns. The total dynamics involved coupled equations of amplitude and phase variables. From the equations of motion, we could estimate the time for transitions between semi-stable states in networks of connected columns showing that the transition rate depended on the connectivity between the columns and also the phase difference. We propose that the transition time reflects a dynamic connectivity measure between columns which is phase dependent and can quickly change value, in fact the possibility exists that the connection can be turned on and off as the dynamical system evolves. There is plenty of support in empirical investigation of cortical activity, measured through micro or macro electrodes, of the idea of a dynamic connectivity. This is often seen in highly non-linear or turbulent activity of the cortex for e.g., the progression of epileptic seizures [21–23]. In the seizure propagation model tested in this paper all connections of the network were positive which would cause the phase difference to quickly tend to 0. This allowed us to simplify the model; however, including a connectivity matrix with a mixture of positive and negative values would cause a complex nodal interaction which was not further analyzed. Investigating the equations of motion revealed that the phase dynamics, at least when perturbed around a semi-stable state, is given by the XY-model (or equivalently the Kuramoto model). Similar proposals of quickly changing connectivity have been presented when analyzing brain activity using the Kuramoto model [20]. Furthermore, there is a vast literature on the dynamics of the XY model, where it has been shown that there is a topological phase transition where the continuous symmetry of the XY-model (or the phase-dynamics presented this study) is broken. The implications for columnar network dynamics would include long range order for the phase variables at low temperatures which breaks at a critical temperature resulting in an exponential decay in phase correlations. This transition has been explained by a change in the topological structure of the dynamics at a critical temperature where low temperature dynamics is governed by stable vortex-antivortex pairs and high temperature activity by free vortices [25].

In contrast to many studies applying the Kuramoto model to cortical activity we have in this paper not assumed it to be true but derived it as a consequence of a network of neural mass models. This strengthens the assumptions of our derivations and furthermore, gives the coupled amplitude dynamics of cortical activity which is usually not associated with the Kuramoto model. The coupling between phase and amplitude dynamics is what allowed us to define *dynamic connectivity*. This was used to generate a model (a forward model) of seizure propagation within a network of cortical columns. Modelling the seizure propagation as a transition between semi-stable states in a network of nodes is novel and has been alluded to in [15] and further elaborated in this paper. This model was compared to simulated neural mass model activity which gave similar findings. Furthermore, we inverted this model using the simulated data to estimate the unknown connectivity matrix. The results did show an improved connectivity matrix, approaching the matrix used to simulate the data; however, this is dependent on the prior estimates and the risk of estimating connectivity matrices at local maxima of the Bayesian free energy, all of which can be nuanced depending on the specific question asked [29]. This part of the paper is a proof of principle of the validity of the seizure progression model that was presented. This model can be further expanded with multifrequency dynamics, which is often seen in clinical data on seizure progression, by including more semi-stable states per cortical column. This study used neural masses to understand the network activity and derive a seizure propagation model. The main reason we chose neural mass models in comparison to other models for cortical activity was for simplicity and practically. Neural field theories for example are more complex and estimating interactions between regions of the cortex is difficult as would be setting up an inversion pathway. One of the main clinical applications of this computational exercise is that we provide a generative model for seizure progression that leads to a relatively straight forward inversion. Furthermore, applying this technique to estimate seizure propagation networks in patient with epilepsy will hopefully provide experimental results that will support the findings of this study. As was discussed shortly in Sect. 3, the cortical layers are approximately decoupled; however, when more experimental results are available the seizure propagation model would allow for a natural complexification of the model by introducing cross layer coupling if needed.

We can now estimate a connectivity matrix from EEG data from seizure progression with a model which 1) specifically models seizure progression 2) is based on the XY-model (equivalently the Kuramoto model) derived from basic neural mass models and 3) biophysically linked to synaptic connectivity. An important clinical question which arises for any patient where possible epilepsy surgery is considered, is to define the optimal region of the brain which, when removed would result in seizure freedom. This can now be modelled prior to surgery by removing or disconnecting parts of the cortical network and estimating the effect that has on seizure propagation. This could be empirically tested using retrospective data on a surgical case series to investigate the possible usefulness of the model and the modifications required to improve applicability to a clinical setting. We have not investigated seizure termination in this study which is of clinical interest as it will define the duration of seizures and hence the clinical impact the seizures will have on a patient. The model we proposed for seizure propagation is a “cascading” network with only unidirectional connectivity, which can model the propagation of seizure activity but not its termination. Using a bi-directional connectivity matrix it is possible to model seizure termination, and the model-inversion should still remain tractable, even though there will be an increase in complexity. This will be further investigated in a future study of seizure propagation and termination.

The equation of motion revealed that the dynamics was only related to phase differences and not the absolute value of phases. The corresponding Hamiltonian for the system would also only be dependent on phase differences between columns. This contrasts with amplitude values which populate freely the equations of motion. The property of phase differences in the Hamiltonian results in excitation levels for the system around a semi-stable equilibrium state of infinitely small energies, i.e., very slowly varying fields [27, 28]. These modes (or goldstone modes) will result in long range correlations between cortical columns. They will contain information of the correlation between a large number of cortical columns. If it is assumed that cortical columns in the healthy brain project information onto semi-stable states, we have now been able to define the process in which the correlations within the total information presented to the brain could be stored. Goldstone modes might be projections of the information processed by the brain; moreover, they are also known to cause the turbulent flow in phase dynamics, something we have suggested might be a key feature in seizure dynamics. We will propose the following hypothesis that cognitive function of the brain and seizure threshold are both dependent on goldstone modes. This could imply that there is a threshold for maximal cognitive function of a brain constructed using cortical columns as the disruption of turbulent phase dynamics is dependent on the same goldstone modes. Interestingly, it is highly probable that epileptic seizures will affect a brain only if it has a minimal complexity; seizures are only known in higher order animals (mammals) [30]. We will in a future study, investigate the implications these low energy excitations have on the dynamics of interconnected cortical columns.

In conclusion we have derived in this paper the dynamics of interconnected cortical columns where the phase and amplitude variables were shown to be the define variables. The equations of motion revealed that excitations of activity around stationary states were governed by the Kuramoto model, a model that has often been used to model phase dynamics of the brain. The results that were derived were applied to construct a forward model for seizure propagation which was inverted using DCM on simulated data. In a future project we will apply this model to actual seizure data investigating the possible clinical impact this might have. Moreover, we will also examine the above theory of cortical connectivity from the perspective of a cortical lattice theory and investigate the implications that would have on modelling cortical function.

## Data Availability

The code and data used in this paper can be obtained from https://github.com/gercoo/sz_propagation.

## Appendix

### Index

The following nomenclature and definitions will be used in this paper.

p_i_ – current variable of the ith population (in layer i) of a cortical column. q_i_ – potential variable of the ith population of a cortical column. z_i_ – complex variable defined using the potential and current term of the ith neuronal population. R_i_ – modulus of z_i_. φ_I_ – phase of z_i_. ω_I_ – natural frequency of the ith neuronal population of a cortical column. ε – Perturbation constant for connectivity via the S-kernel. μ – Perturbation constant for connectivity via P-kernel. R_i,n,m_ – term with coefficient ε^n^μ^m^ in the perturbative series expansion of R_i_. φ_i,n,m_ – term with coefficient ε^n^μ^m^ in the perturbative series expansion of φ_i_. S – A synaptic kernel mapping potentials to currents (given by tanh). S_r_ – rth order term in the Taylor series of S around 0. P – A synaptic kernel mapping currents to currents (for spike rate adaptability). P_r_ – rth order term in the Taylor series of P around 0. s_ij_ – Connectivity gain between the ith and jth population via a type S synaptic kernel. p_ij_ – Connectivity gain between the ith and jth population via a type P synaptic kernel. h_ij_ – Connectivity gain between the ith population in a cortical-columns and the jth population in a second cortical column (these connections could be either type S or type P connections).

### Adiabatic approximation of neural mass model

The neural mass model will be given by the following complex ordinary differential equation. Sums in the equations run over the layers of a cortical column (roman indexes). Each equation will give the activity of one layer of the cortical column. The interactions between the layers are via the synaptic kernels S and P. The S-synaptic kernel is given by a sigmoid function (tanh). The P-synaptic kernel will be assumed to have several 0 points (allowing for multiple semi-stable states).

5.2.1 $${\dot{z}}_{i}=-i{\omega }_{i}{z}_{i}+i\varepsilon {\omega }_{i}\sum {s}_{ij}S\left(\frac{{z}_{j}+{{z}_{j}}^{*}}{2}\right)+i\mu {\omega }_{i}\sum {p}_{ij}P\left(\frac{{z}_{j}-{{z}_{j}}^{*}}{2i}\right)$$

The complex trajectory function, *z*, can be written in modulus-argument form,

$${z}_{i}(t)={R}_{i}\left(t,\varepsilon ,\mu \right){e}^{-i({\omega }_{i}t+{\varphi }_{i}(\left(t,\varepsilon ,\mu \right)))}$$

The adiabatic approximation can be done analytically if the perturbation constants are small, which will be assumed. A perturbative series expansion of the equations of motion in terms of ε and μ was done using the following expansions,

$$S\left(\frac{{z}_{j}+{{z}_{j}}^{*}}{2}\right)= \sum_{r=1}^{\infty }{S}_{r}{\left(\frac{{z}_{j}+{{z}_{j}}^{*}}{2}\right)}^{2r-1}$$

$$P\left(\frac{{z}_{j}-{{z}_{j}}^{*}}{2i}\right)= \sum_{r=1}^{\infty }{P}_{r}{\left(\frac{{z}_{j}-{{z}_{j}}^{*}}{2i}\right)}^{2r-1}$$

$${R}_{i}\left(t,\varepsilon ,\mu \right)={R}_{i,\mathrm{0,0}}+\sum {\varepsilon }^{m}{\mu }^{n}{R}_{i,m,n}(t)$$

$${\varphi }_{i}\left(t,\varepsilon ,\mu \right)={\varphi }_{i,\mathrm{0,0}}+\sum {\varepsilon }^{m}{\mu }^{n}{\varphi }_{i,m,n}(t)$$

Using these expansions, the left-hand side (LHS) of the equation of motion (Eq. 5.2.1) will be given by the following equation.

$$LHS=\left[\sum_{k=0}^{\infty }\frac{{\left(-i{\varphi }_{i}\right)}^{k}}{k!}\right]\left(\sum {\varepsilon }^{n}{\mu }^{m}{\dot{R}}_{i,n,m}\left(t\right)\right){e}^{-i{\omega }_{i}t}-i\left({R}_{i,\mathrm{0,0}}+\sum {\varepsilon }^{n}{\mu }^{m}{R}_{i,n,m}\left(t\right)\right)*\left({\omega }_{i}+\sum {\varepsilon }^{n}{\mu }^{m}{\dot{\varphi }}_{i,n,m}(t)\right)\left[\sum_{k=0}^{\infty }\frac{{\left(-i{\varphi }_{i}\right)}^{k}}{k!}\right]{e}^{-i{\omega }_{i}t}$$

The right-hand side (RHS) of the equation of motion (Eq. 5.2.1) will be given by the following equation.

$$RHS= -i{\omega }_{i}\left[\sum_{k=0}^{\infty }\frac{{\left(-i{\varphi }_{i}\right)}^{k}}{k!}\right]\left({R}_{i,\mathrm{0,0}}+\sum {\varepsilon }^{n}{\mu }^{m}{R}_{i,n,m}\left(t\right)\right){e}^{-i{\omega }_{i}t}+i{\omega }_{i}\varepsilon \sum {s}_{ij}\sum_{r=1}^{\infty }{S}_{r}\frac{{\left({R}_{j,\mathrm{0,0}}+\sum {\varepsilon }^{n}{\mu }^{m}{R}_{j,n,m}(t)\right)}^{2r-1}}{{2}^{2r-1}}*\left[\sum_{l=0}^{2r-1}\left(\begin{array}{c}2r-1\\ l\end{array}\right){e}^{-i\left(\left(2r-1\right)-2l\right)\left({\omega }_{j}t\right) }\left[\sum_{k=0}^{\infty }\frac{{\left(-i(2r-1-2l){\varphi }_{j}\right)}^{k}}{k!}\right]\right]+i{\omega }_{i}\mu \sum {p}_{ij}\sum_{r=1}^{\infty }{(-1)}^{r}{P}_{r}\frac{{\left({R}_{j,\mathrm{0,0}}+\sum {\mu }^{n}{R}_{j,0,n}(t)\right)}^{2r-1}}{{2}^{2r-1}}*\left[\sum_{l=0}^{2r-1}{(-1)}^{l}\left(\begin{array}{c}2r-1\\ l\end{array}\right){e}^{-i\left(\left(2r-1\right)-2l\right)\left({\omega }_{j}t\right) }\left[\sum_{k=0}^{\infty }\frac{{\left(-i(2r-1-2l){\varphi }_{j}\right)}^{k}}{k!}\right]\right]$$

An adiabatic approximation was done over a complete cycle of oscillation of the cortical column. The changes in the trajectory after integrating over a full cycle (defined subsequently as the derivative of the flow in the new time scale) showed that the amplitude variable was only affected by *P*-coupling (first order expansion terms) and combinations of *P*- and *S*-coupling (2nd order expansion terms) as shown below,

5.2.2 $$\frac{d{R}_{i}}{dt}=\mu \frac{d{R}_{i,\mathrm{0,1}}}{dt}+\mu \varepsilon \frac{d{R}_{i,\mathrm{1,1}}}{dt}$$

The amplitude terms of Eq. (5.5.5) will be given by the following expressions,

$$\frac{d{R}_{i,\mathrm{0,1}}}{dt}={\omega }_{i}{p}_{ii}\sum_{r=1}^{\infty }{P}_{r}\frac{{{R}_{i,\mathrm{0,0}}}^{2r-1}}{{2}^{2r-1}}\left(\begin{array}{c}2r-1\\ r-1\end{array}\right)$$

$$\frac{d{R}_{i,\mathrm{1,1}}}{dt}=\sum \frac{{s}_{ij}{p}_{ji}{\omega }_{i}{\omega }_{j}}{{{\omega }_{j}}^{2}-{{\omega }_{i}}^{2}}*\sum_{r,s=1}^{\infty }\left[\left(r-1\right){S}_{r}{P}_{s}{\omega }_{j}+r{S}_{s}{P}_{r}{\omega }_{i}\right]\frac{{{R}_{j,0}}^{2r-2}{{R}_{i,\mathrm{0,0}}}^{2s-1}}{{2}^{2r+2s-3}}\left(\begin{array}{c}2r-1\\ r\end{array}\right)\left(\begin{array}{c}2s-1\\ s\end{array}\right)$$

The other terms in the perturbation series up to 2nd order cancel or are smaller than the given terms (i.e., μ^2^, ε, ε^2^). Equation (5.2.2) was used to plot the dynamics of a cortical column with 2 layers (Fig. 4) with the following variables,

ω_1_ = 1. ω_2_ = 2.

s_12_ = p_12_ = s_21_ = p_21_ = 0 or 0.1. p_11_ = p_22_ = 0 or 0.1. S(x) = tanh(x).

P – was defined using a Taylor series with the following coefficients:

P_1_ = − 0.1. P_2_ = 1.5. P_3_ = − 3.2

#### Interaction between 2 cortical columns

The dynamics of two cortical columns will be examined by connecting the two columns using extrinsic (extra-columnar) connections. The intrinsic connections (intracolumnar) will be denoted by g_ij_, and the extrinsic will be denoted by h_ij_. The equations determining the motion of the 2 cortical columns will be given by the following equations (one for each column). Different cortical columns are indexed by superscripts.

5.3.1 $$\left({\dot{R}}_{i}^{1}\left(t,\varepsilon ,\mu \right){e}^{-i\left({\omega }_{i}t+{\varphi }_{i}^{1}\right)}-i{R}_{i}^{1}\left(t,\varepsilon ,\mu \right){\dot{\varphi }}_{i}^{1}{e}^{-i\left({\omega }_{i}t+{\varphi }_{i}^{1}\right)}\right)=i\varepsilon {\omega }_{i}\sum {g}_{ij}S\left(\frac{{z}_{j}^{1}+{{z}_{j}^{1}}^{*}}{2}\right)+i\mu {\omega }_{i}\sum {g}_{ij}P\left(\frac{{z}_{j}^{1}-{{z}_{j}^{1}}^{*}}{2i}\right)+i\varepsilon {\omega }_{i}\sum {h}_{ij}S\left(\frac{{z}_{j}^{2}+{{z}_{j}^{2}}^{*}}{2}\right)+i\mu {\omega }_{i}\sum {h}_{ij}P\left(\frac{{z}_{j}^{2}-{{z}_{j}^{2}}^{*}}{2i}\right)$$

As was shown in Sect. 5.2, the 2nd, 3rd and 4th term on the RHS of Eq. (5.3.1) will affect the phase portrait topology, but the 1st term will not. The 3rd and 4th term will affect the phase space structure due to the phase difference between the two cortical columns. After integrating over a full cycle (the adiabatic approximation) we get the following expressions,

$$i\varepsilon {\omega }_{i}\sum {g}_{ij}S\left(\frac{{z}_{j}^{1}+{{z}_{j}^{1}}^{*}}{2}\right)\to -i\varepsilon {\omega }_{i}{g}_{ii}\sum_{r=1}^{\infty }{S}_{r}\frac{{{R}_{i}^{1}}^{2r-1}}{{2}^{2r-1}}\left(\begin{array}{c}2r-1\\ r-1\end{array}\right)$$

$$i\mu {\omega }_{i}\sum {g}_{ij}P\left(\frac{{z}_{j}^{1}-{{z}_{j}^{1}}^{*}}{2i}\right)\to \mu {\omega }_{i}{g}_{ii}\sum_{r=1}^{\infty }{P}_{r}\frac{{{R}_{i}^{1}}^{2r-1}}{{2}^{2r-1}}\left(\begin{array}{c}2r-1\\ r-1\end{array}\right)$$

$$i\varepsilon {\omega }_{i}\sum {h}_{ij}S\left(\frac{{z}_{j}^{2}+{{z}_{j}^{2}}^{*}}{2}\right)\to i\varepsilon {\omega }_{i}{h}_{ii}\sum_{r=1}^{\infty }{S}_{r}\frac{{{R}_{i}^{2}}^{2r-1}}{{2}^{2r-1}}\left(\begin{array}{c}2r-1\\ r-1\end{array}\right){e}^{i\left({\varphi }_{i}^{1}-{\varphi }_{i}^{2}\right)}$$

$$i\mu {\omega }_{i}\sum {h}_{ij}P\left(\frac{{z}_{j}^{2}-{{z}_{j}^{2}}^{*}}{2i}\right)\to \mu {\omega }_{i}{h}_{ii}\sum_{r=1}^{\infty }{P}_{r}\frac{{{R}_{i,\mathrm{0,0}}^{2}}^{2r-1}}{{2}^{2r-1}}\left(\begin{array}{c}2r-1\\ r-1\end{array}\right){e}^{i({\varphi }_{i}^{1}-{\varphi }_{i}^{2})}$$

The amplitude and phase dynamics will be a sum of the above equations depending on the types of connections present between the cortical columns. We get the following relation assuming only connections of P-type,

5.3.2 $$\frac{d}{dt}{R}_{i}^{1}={\omega }_{i}{g}_{ii}\sum_{r=1}^{\infty }{P}_{r}\frac{{{R}_{i}^{1}}^{2r-1}}{{2}^{2r-1}}\left(\begin{array}{c}2r-1\\ r-1\end{array}\right)+{\omega }_{i}{h}_{ii}{\text{cos}}({\varphi }_{i}^{1}-{\varphi }_{i}^{2})\sum_{r=1}^{\infty }{P}_{r}\frac{{{R}_{i}^{2}}^{2r-1}}{{2}^{2r-1}}\left(\begin{array}{c}2r-1\\ r-1\end{array}\right)$$

$$\frac{d}{dt}{\varphi }_{i}^{1}=-{\omega }_{i}{h}_{ii}{\text{sin}}({\varphi }_{i}^{1}-{\varphi }_{i}^{2})\sum_{r=1}^{\infty }{P}_{r}\frac{{{R}_{i}^{2}}^{2r-1}}{{R}_{i}^{1}{2}^{2r-1}}\left(\begin{array}{c}2r-1\\ r-1\end{array}\right)$$

### Phase dynamics

The phase lag and amplitude dynamics are coupled; however, as an approximation (mainly to keep the results tractable) we will uncouple them by using the average amplitude values (averaged over different trajectories when the system is under a noise process)

$${R}_{i}^{k}\to \langle {R}_{i}^{k}\rangle$$

The phase dynamics when both cortical columns are in the high amplitude state will be given by the following equation which is equiavalent to the Kuramoto model.

5.4.1 $$\frac{d}{dt}{\varphi }_{i}^{1}=-{\omega }_{i}{h}_{ii}{\text{sin}}({\varphi }_{i}^{1}-{\varphi }_{i}^{2})\sum_{r=1}^{\infty }{B}_{r}\frac{{\langle {R}_{i}^{2}\rangle }^{2r-1}}{\langle {R}_{i}^{1}\rangle {2}^{2r-1}}\left(\begin{array}{c}2r-1\\ r-1\end{array}\right)$$

This equation will approximate the activity around the stationary sets (stationary point or limit cycles) of the cortical columns. It is equivalent to the celebrated Kuramoto model when connected to a cortical column in a high amplitude state.

### Transition from near zero state to high amplitude state

The transition time between the two semi-stable states (near 0 to high amplitude state) when under the influence of a second cortical column in a high amplitude state can be estimated using Eq. (5.3.2). Integrating this equation along R_1_ will give the potential trapping the trajectories near the stable points (A is a constant).

$$U=-{\omega }_{i}{g}_{ii}\sum_{r=1}^{\infty }{B}_{r}\frac{{{R}_{i}^{1}}^{2r}}{r{2}^{2r}}\left(\begin{array}{c}2r-1\\ r-1\end{array}\right)-A{\omega }_{i}{h}_{ii}{\text{cos}}({\varphi }_{i}^{1}-{\varphi }_{i}^{2}){R}_{i}^{1}$$

The Fokker Planck equation resulting from the flow of the above gradient with a noise process would be given by the following expression (amplitude of the noise process will be denoted by σ_n_ and the Fokker Planck operator will be denoted L_FP_). The probability distribution is given by ρ.

5.5.1 $$\frac{\partial \rho }{\partial t}=-\frac{\partial }{\partial x}\left(\rho \left(\frac{\partial }{\partial x}U\right)\right)+\frac{{{\sigma }_{n}}^{2}}{2}\frac{{\partial }^{2}\rho }{\partial {x}^{2}}={L}_{FP}(\rho )$$

$$\partial \rho ={L}_{FP}(\rho )\partial t$$

This Fokker Planck flow will be projected to the space of normal distributions. The flow of the distributions could then be parameterised using the mean (μ) and the standard deviation (σ) of the true distribution. To simplify the integration, we will assume integration over the whole real line.

$$\int x\left(\rho + \partial \rho \right)-\int x\rho =d\mu$$

$$\frac{d\mu }{dt}=\int x\frac{\partial \rho }{\partial t}$$

$$\int {x}^{2} \partial \rho -2\int x\partial \rho ={\left(\sigma +d\sigma \right)}^{2}-{\sigma }^{2}=2\sigma d\sigma$$

$$\int \left({x}^{2}-2x\right) \frac{\partial \rho }{\partial t}=\frac{2\sigma d\sigma }{dt}$$

The last term of the Fokker Planck eq., the diffusion component, will not affect the mean but will have a component for the change in standard deviation.

$$\int \left({x}^{2}-2x\right) \frac{\partial \rho }{\partial t}=\int {x}^{2} \frac{\partial \rho }{\partial t}=\frac{2\sigma d\sigma }{dt}$$

$$\frac{{{\sigma }_{n}}^{2}}{2}=\frac{\sigma d\sigma }{dt}$$

$${\sigma }^{2}={{\sigma }_{n}}^{2}t$$

The drift term of the FP-eq will give the following,

$$-\frac{\partial }{\partial x}\left(\rho \frac{\partial }{\partial x}U\right)={\omega }_{i}{g}_{ii}\sum_{r=1}^{\infty }{P}_{r}\left[\rho \left(2r-1\right)\frac{{x}^{2r-2}}{{2}^{2r-1}}+\frac{d\rho }{dx}\frac{{x}^{2r-1}}{{2}^{2r-1}}\right]\left(\begin{array}{c}2r-1\\ r-1\end{array}\right)+{\omega }_{i}{h}_{ii}\frac{d\rho }{dx}{\text{cos}}({\varphi }_{i}^{1}-{\varphi }_{i}^{2}){A}_{1}$$

$$\frac{d\mu }{dt}=\int x\frac{\partial \rho }{\partial t}=\int {\omega }_{i}{g}_{ii}\sum_{r=1}^{\infty }{P}_{r}\left[\rho \left(2r-1\right)\frac{{x}^{2r-1}}{{2}^{2r-1}}+\frac{d\rho }{dx}\frac{{x}^{2r}}{{2}^{2r-1}}\right]\left(\begin{array}{c}2r-1\\ r-1\end{array}\right)+{\omega }_{i}{h}_{ii}x\frac{d\rho }{dx}{\text{cos}}({\varphi }_{i}^{1}-{\varphi }_{i}^{2})C$$

$$\rho =\frac{1}{\sigma \sqrt{2\pi }}{\text{exp}}\left(-{\frac{1}{2}\left(\frac{x-\mu }{\sigma }\right)}^{2}\right)$$

$$\frac{d\rho }{dx}=-\frac{1}{{\sigma }^{2}\sqrt{2\pi }}\left(\frac{x-\mu }{\sigma }\right){\text{exp}}\left(-{\frac{1}{2}\left(\frac{x-\mu }{\sigma }\right)}^{2}\right)$$

We will change the variable of integration as follows,

$$z=\frac{x-\mu }{\sigma }$$

$$\sigma dz=dx$$

$$dx\rho \left(x\right)=\frac{dz}{\sqrt{2\pi }}{\text{exp}}\left(-\frac{1}{2}{z}^{2}\right)=dzp(z)$$

$$dx\frac{d\rho }{dx}=-\frac{dz}{\sigma \sqrt{2\pi }}z{\text{exp}}\left(-\frac{1}{2}{z}^{2}\right)$$

$$\frac{d\mu }{dt}=\int {\omega }_{i}{g}_{ii}\sum_{r=1}^{\infty }{P}_{r}\left[\rho \left(2r-1\right)\frac{{(\sigma z+\mu )}^{2r-1}}{{2}^{2r-1}}+\frac{d\rho }{dx}\frac{{(\sigma z+\mu )}^{2r}}{{2}^{2r-1}}\right]\left(\begin{array}{c}2r-1\\ r-1\end{array}\right) +{\omega }_{i}{h}_{ii}(\sigma z+\mu )\frac{d\rho }{dx}{\text{cos}}({\varphi }_{i}^{1}-{\varphi }_{i}^{2})C$$

$$\begin{aligned} \smallint dz\rho \left( {\sigma z + \mu } \right)^{2r - 1} = & \mathop \sum \limits_{s = 0}^{r - 1} \left( {\begin{array}{*{20}c} {2r - 1} \\ {2s} \\ \end{array} } \right)\sigma^{2s} \mu^{2r - 1 - 2s} \smallint z^{2s} \rho \\ & \left( {\smallint z^{2s} \rho = \left( {2s - 1} \right)!!} \right) \\ \end{aligned}$$

$$\smallint dz\rho \left( {\sigma z + \mu } \right)^{2r - 1} = \mathop \sum \limits_{s = 0}^{r - 1} \left( {\begin{array}{*{20}c} {2r - 1} \\ {2s} \\ \end{array} } \right)\sigma^{2s} \mu^{2r - 1 - 2s} \left( {2s - 1} \right)!!$$

$$\int dx\frac{d\rho }{dx}{(\sigma z+\mu )}^{2r}=-\frac{1}{\sigma }\int dzz\rho {\left(\sigma z+\mu \right)}^{2r}=-\sum_{s=1}^{r}\frac{1}{\sigma }\left(\begin{array}{c}2r\\ 2s-1\end{array}\right){\sigma }^{2s-1}{\mu }^{2r-2s+1}\int {z}^{2s}\rho$$

$$\smallint dx\frac{d\rho }{{dx}}\left( {\sigma z + \mu } \right)^{2r} = - \mathop \sum \limits_{s = 1}^{r} \left( {\begin{array}{*{20}c} {2r} \\ {2s - 1} \\ \end{array} } \right)\sigma^{2s - 2} \mu^{2r - 2s + 1} \left( {2s - 1} \right)!!$$

$$\int dx(\sigma z+\mu )\frac{d\rho }{dx}=-\frac{1}{\sigma }\int dzz\rho \left(\sigma z+\mu \right)=-\int dz{z}^{2}\rho =-1$$

We then have for the full integral,

$$\begin{aligned} \frac{d\mu }{{dt}} = & \omega_{i} g_{ii} \mathop \sum \limits_{r = 1}^{\infty } B_{r} \left[ {\left( {2r - 1} \right)\frac{{\mathop \sum \nolimits_{s = 0}^{r - 1} \left( {\begin{array}{*{20}c} {2r - 1} \\ {2s} \\ \end{array} } \right)\sigma^{2s} \mu^{2r - 1 - 2s} \left( {2s - 1} \right)!!}}{{2^{2r - 1} }}} \right. \\ & - \left. {\frac{{\mathop \sum \nolimits_{s = 1}^{r} \left( {\begin{array}{*{20}c} {2r} \\ {2s - 1} \\ \end{array} } \right)\sigma^{2s - 2} \mu^{2r - 2s + 1} \left( {2s - 1} \right)!!}}{{2^{2r - 1} }}} \right]\left( {\begin{array}{*{20}c} {2r - 1} \\ {r - 1} \\ \end{array} } \right) - \omega_{i} h_{ii} {\text{cos}}\left( {\varphi_{i}^{1} - \varphi_{i}^{2} } \right)C \\ \end{aligned}$$

Simplifying if ${h}_{ii}>{g}_{ii}$, we get the following expression for the change in mean of the trajectory.

$$\frac{d\mu }{dt}=-{\omega }_{i}{h}_{ii}{\text{cos}}\left({\varphi }_{i}^{1}-{\varphi }_{i}^{2}\right)C\propto -{h}_{\mathit{ii}}{\text{cos}}\left({\varphi }_{i}^{1}-{\varphi }_{i}^{2}\right)$$

Which in turn could be integrated into the following expression for the mean (μ) parameter,

5.5.2 $$\mu \left(t\right)=\mu \left(0\right)-{h}_{\mathit{ii}}{\text{cos}}\left({\varphi }_{i}^{1}-{\varphi }_{i}^{2}\right)t$$

As a typical example we will simulate seizure onset times (*T*_*j*_) for N electrodes with an unknown connectivity matrix which will be estimated using the onset times and phase lag values between each electrode pair. The mean of the distribution of the ith population can be approximated (after absorbing constants into the definition of h) using the following equation (Θ is the Heaviside step function).

$${\mu }_{i}\left(t\right)={\mu }_{i}\left(0\right)-\sum_{j<i}^{N}{h}_{\mathit{ji}}{\text{cos}}\left({\varphi }_{i}-{\varphi }_{j}\right){\int }_{0}^{t}dt{\Theta }_{j}(t{\prime}-{T}_{j})$$

$${\Theta }_{j}\left({t}{\prime}-{T}_{j}\right)\approx \frac{1}{2}+\frac{1}{2}{\text{tanh}}({t}{\prime}-{T}_{j})$$

$${\int }_{0}^{t}dt\frac{1}{2}+\frac{1}{2}{\text{tanh}}({t}{\prime}-{T}_{j})=\frac{t}{2}+\frac{1}{2}{\text{ln}}\left({\text{cosh}}\left({t}{\prime}-{T}_{j}\right)\right)-\frac{1}{2}{\text{ln}}\left({\text{cosh}}\left({T}_{j}\right)\right)$$

$${\mu }_{i}\left(t\right)={\mu }_{i}\left(0\right)-\sum_{j<i}^{N}{h}_{\mathit{ji}}{\text{cos}}\left({\varphi }_{i}-{\varphi }_{j}\right)\left[\frac{t}{2}+\frac{1}{2}{\text{ln}}\left({\text{cosh}}\left(t-{T}_{j}\right)\right)-\frac{1}{2}{\text{ln}}\left({\text{cosh}}\left({T}_{j}\right)\right)\right]$$

We will take ${\mu }_{i}\left(0\right)=0$ and so that the transition time will be defined by the following equation.

$$0=1+\sum_{j<i}^{N}{h}_{\mathit{ji}}{\text{cos}}\left({\varphi }_{i}-{\varphi }_{j}\right)\left[\frac{{T}_{i}}{2}+\frac{1}{2}{\text{ln}}\left({\text{cosh}}\left({T}_{i}-{T}_{j}\right)\right)-\frac{1}{2}{\text{ln}}\left({\text{cosh}}\left({T}_{j}\right)\right)\right]={F}_{i}(\overrightarrow{T})$$

$${\mu }_{i}\left(T\right)=1$$

The solution to the following equation will estimate onset times as a function of connectivity, ***h***.

$$\overrightarrow{F}\left(\overrightarrow{T}\right)=0$$

The dynamics is determined by the connectivity matrix, ***h***, and phase lag between the cortical columns.

### Summary of dynamic causal modelling

We provide a short summary and references for the inversion scheme used in this study. Please see the following publications for a description of the inversion scheme [31–33]. One of the main reasons in performing the DCM is to estimate a posterior distribution for the parameters of the model given the measured data. In the present study it would be the unknown connectivity matrix. The inversion requires defining a forward model which will be a function of the unknown parameters mapping to the data space. The forward model will in this case give the likelihood of the data given fixed parameters.

$$f\left(x|\theta \right)$$

To estimate the posterior distributions of the parameters given the data, Bayes theorem will be used.

$$p\left(\theta |x\right)\propto f\left(x|\theta \right)p\left(\theta |h\right)$$

Both the prior and posterior distributions are assumed to be Gaussian (Laplace approximation). As the forward model is non-linear, several iterations of the estimation of the posterior are required and is done using the Expectation-Maximation algorithm (EM) [34]. In the E-step the conditional expectations and covariances of the parameters are estimated. This is then followed by the M-step where the hyperparameters (mean and covariance of the prior distribution) are estimated. This is done repeatedly until the change in free energy of the estimated model is smaller than a given tolerance level.
